# Catheter ablation in patients with persistent atrial fibrillation

**DOI:** 10.1093/eurheartj/ehw260

**Published:** 2016-07-07

**Authors:** Paulus Kirchhof, Hugh Calkins

**Affiliations:** 1 Institute of Cardiovascular Sciences, University of Birmingham, IBR, Room # 136, Birmingham B15 2TT, UK; 2 SWBH and UHB NHS Trusts, Birmingham, UK; 3 Atrial Fibrillation NETwork (AFNET), Münster, Germany; 4 Department of Cardiovascular Medicine, Hospital of the University of Münster, Münster, Germany; 5 Johns Hopkins University, Baltimore, MD, USA

**Keywords:** Atrial fibrillation, Persistent, Long-standing persistent, Catheter ablation, Technique, Indications, Outcomes, Sinus rhythm, Weight loss, Exercise, Complications, Clinical practice, Antiarrhythmic drugs, Rhythm control therapy, Upstream therapy

## Abstract

Catheter ablation is increasingly offered to patients who suffer from symptoms due to atrial fibrillation (AF), based on a growing body of evidence illustrating its efficacy compared with antiarrhythmic drug therapy. Approximately one-third of AF ablation procedures are currently performed in patients with persistent or long-standing persistent AF. Here, we review the available information to guide catheter ablation in these more chronic forms of AF. We identify the following principles: Our clinical ability to discriminate paroxysmal and persistent AF is limited. Pulmonary vein isolation is a reasonable and effective first approach for catheter ablation of persistent AF. Other ablation strategies are being developed and need to be properly evaluated in controlled, multicentre trials. Treatment of concomitant conditions promoting recurrent AF by life style interventions and medical therapy should be a routine adjunct to catheter ablation of persistent AF. Early rhythm control therapy has a biological rationale and trials evaluating its value are underway. There is a clear need to generate more evidence for the best approach to ablation of persistent AF beyond pulmonary vein isolation in the form of adequately powered controlled multi-centre trials.

## Introduction

Recent prevalence estimates suggest that at least 33.5 million persons are affected by atrial fibrillation (AF).^1^ Catheter ablation is increasingly offered to relieve AF-related symptoms,^2–4^ based on evidence illustrating its efficacy compared with antiarrhythmic drug therapy.^5–9^ There is less evidence supporting AF ablation in persistent AF, although small studies suggest better maintenance of sinus rhythm. Two years ago, the first multicentre trial comparing catheter ablation with cardioversion and antiarrhythmic drugs as first-line therapy for persistent AF has been reported and demonstrated more effective maintenance of sinus rhythm, as well as better quality of life, in patients randomized to catheter ablation.^10^ In recently published surveys, approximately one-third of AF ablation procedures were performed in patients with persistent or long-standing persistent AF.^4^ Here, we discuss recent data suggesting that our clinical ability to discriminate paroxysmal and persistent AF is limited, review the evidence supporting the use of catheter ablation in persistent AF, illustrate approaches to improve sinus rhythm maintenance by comprehensive cardiovascular risk reduction,^11^ discuss the value of different ablation strategies, and highlight the need for adequate validation of novel approaches to catheter ablation for persistent AF.

## What is persistent atrial fibrillation

Persistent AF is defined as AF that persists without interruption for 7 days or longer.^5,6^ Whether patients who have been cardioverted during the first 7 days of an AF episode should be classified as persistent or paroxysmal has been defined differently in the USA and in Europe,^5,6^ but this only pertains to a small number of patients who receive early cardioversion. Patients who are in AF for >1 year are classed as long-standing persistent AF.^5,6^ Most patients who initially present with paroxysmal, self-terminating AF will progress to chronic forms of the arrhythmia (including permanent AF) or switch back and forth between paroxysmal and persistent or long-standing persistent AF.^12,13^ This simple observation already suggests that paroxysmal and persistent AF are not biologically distinct entities, but rather constitute different presentations of the same arrhythmia, loosely associated with different stages of the disease. Consistent with this general concept, patients diagnosed with persistent AF are generally older than those in paroxysmal AF, and present with more comorbidities.^14,15^ Over the last decade, the ECG-monitoring technology available to health care professionals and to the general public has tremendously advanced, thus profoundly improving our ability to differentiate AF patterns, reflected in consensus statements that informed regulatory bodies.^7,16^ Importantly, a recent analysis of continued atrial rhythm monitoring using implanted devices suggests that the AF pattern (or ‘AF burden’) does not differ too much between patients who have been clinically diagnosed with ‘paroxysmal’ or ‘persistent’ AF.^17^ Hence, the mere clinical classification of the AF pattern as ‘persistent’ may neither be sufficient justification to decide on a specific ablation strategy nor too powerful to predict the effectiveness of catheter ablation. It rather seems reasonable to assume that some patients with persistent AF respond to pulmonary vein isolation (PVI) as well as patients in paroxysmal AF, while others (with either AF pattern) are likely to develop recurrent AF.

## Prevention of recurrent atrial fibrillation in patients scheduled for ablation of persistent atrial fibrillation

**Figure 1:**
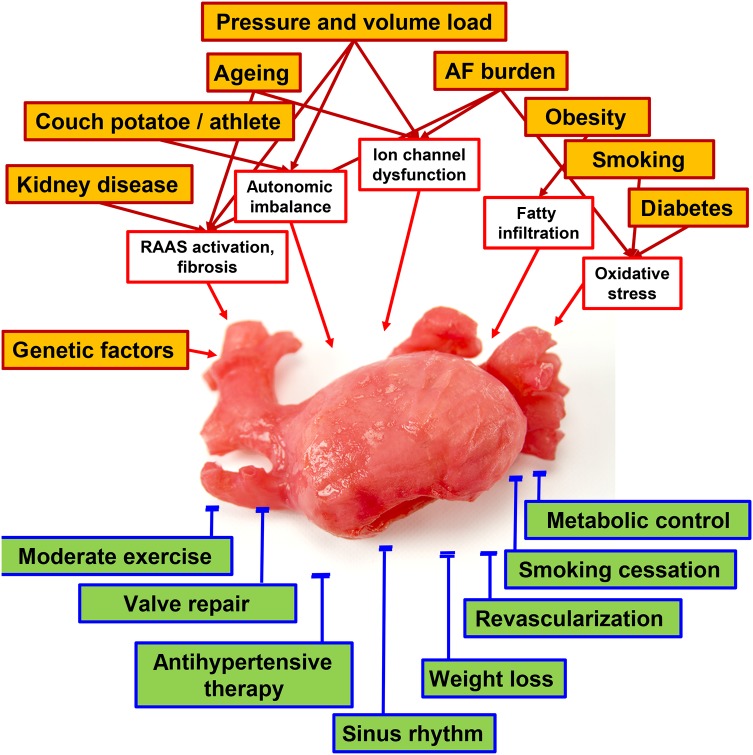
Major health modifiers promoting recurrent atrial fibrillation (orange boxes) and the likely intermediary mechanisms causing atrial damage and leading to atrial fibrillation (open boxes, top part, health modifiers taken from Fabritz *et al.*^89^). The green boxes at the bottom illustrate interventions that can mitigate or reverse these effects. These ancillary interventions should be an integral part of the management of patients undergoing catheter ablation of persistent atrial fibrillation.

It is well established that many patients already present with substantial atrial damage at the first episode of AF, and concomitant conditions such as hypertension, obesity, heart failure, chronic kidney disease, and others will cause ‘remodelling’ of the atria prior to occurrence of AF.^11,18^ On top of this ‘remodelling’, AF induces electrical and structural changes in the atria that occur within days to weeks (*Figure 1*). Nonetheless, some patients develop AF without detectable concomitant conditions late in life.^19^

A number of factors promote recurrent AF in patients, including those that undergo ablation of AF. Interestingly, time since the first diagnosis of AF is only a weak factor, while the time spent in continuous AF prior to ablation is a better predictor of outcome, where maintenance of sinus rhythm after ablation is less likely in patients spending >3 years continuously in AF prior to ablation (‘long-standing persistent’ AF).^20^

Other drivers of recurrent AF are related to clinical conditions that promote structural remodelling. Left atrial remodelling, which can be assessed invasively by quantifying areas of low-voltage left atrial signals, or with MR imaging,^21–24^ is largely determined by concomitant cardiovascular conditions and possibly by duration of AF, while other factors were derived from clinical information.^25,26^ It is worthwhile to consider that the effect of any AF ablation procedure is to induce further areas of delayed gadolinium enhancement.^23,27^

While the natural ageing process is presently difficult to modulate, several factors associated with recurrent AF after catheter ablation can be modified by medical therapy or life style interventions (*Table 1*). Initial results of such life style interventions, e.g. regular exercise^28^ or systematic weight reduction in obese patients scheduled for AF ablation,^29,30^ are promising. Initial results also suggest that antihypertensive therapies that modulate central sympathetic tone (moxonidine) reduce recurrent AF after catheter ablation.^31^ Angiotensin converting enzyme inhibitors or sartans, while not effective in preventing recurrent AF in patients without structural heart disease in the short term,^32,33^ may have long-term beneficial effects for the primary prevention of AF.^34^ It has also been suggested that evidence-based therapy of heart failure with reduced ejection fraction can help to prevent AF.^35^ Likewise, adequate revascularization and treatment of mitral valve disease are likely to help stabilize sinus rhythm and restore atrial function (*Figure 1*). While it will be difficult to evaluate each of these interventions separately in outcome trials, it seems reasonable to integrate adequate treatment of modifiable cardiovascular conditions into the management of patients undergoing ablation of AF based on their known general cardiovascular benefits.^36^ In addition, it is common practice to use antiarrhythmic drugs for 3 months after catheter ablation of AF, including for persistent AF. Such treatment probably suppresses short-term recurrences of AF, but does not alter the mid-term recurrence rate.^37^ This practice seems reasonable.

**Table 1:** Clinical factors that contribute to recurrent atrial fibrillation after catheter ablation and potential interventions that could reduce their impact on recurrent atrial fibrillation

Factor associated with recurrent AF	Possible intervention
Age	None available
Chronic kidney disease	?
Diabetes	Weight reduction, regular exercise (?)
Obesity	Weight reduction, regular exercise
Hypertension	Antihypertensive therapy, possibly including monoxidine and RAAS inhibition
Heart failure	Therapy of HFrEF with ACE inhibitors, β blockers, mineralocorticoid antagonists, etc.
High ventricular rate	Rate control therapy (?)
Left atrial diameter	None available
Duration of continuous AF prior to ablation	Early rhythm control therapy (?)

ACE, angiotensin converting enzyme; AF, atrial fibrillation; HFrEF, heart failure with reduced ejection fraction; RAAS, renin–angiotensin aldosterone.

**Figure 2:**
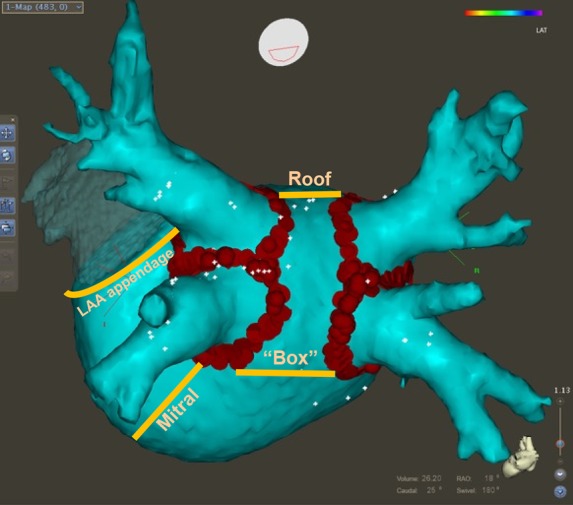
Reconstruction of the left atrium (posterior view) showing the pulmonary veins and the left atrial appendage. Red dots illustrate the current approach of isolation of the pulmonary veins, in this case including a line between the two superior and inferior veins. Orange lines indicate additional linear ablation lesions that have been proposed to enhance the success rate of atrial fibrillation ablation (roof line, mitral isthmus line, ‘box’ lesions consisting of a roof/superior and inferior connection between the pulmonary vein isolation circles, and left atrial appendage isolation). The effectiveness of these additional ablation interventions will require evaluation in adequately sized and powered controlled trials.

## Pulmonary vein isolation prevents recurrent atrial fibrillation in some, but not all patients with persistent atrial fibrillation

The first description that triggers in the pulmonary veins initiate AF and that their elimination by radio frequency ablation prevents AF has been a key disruptive discovery shaping AF ablation.^38^ Pulmonary vein isolation remains the cornerstone of AF ablation until today.^7^ Two-thirds of surveyed European centres perform PVI without additional ablation targets as a first-line therapy for persistent AF.^39^ Pulmonary vein isolation conveys a 60–80% rate of maintaining sinus rhythm after 1 year in patients who predominantly present with paroxysmal forms of AF, not different between centres mainly using cryoballoon and irrigated radio frequency ablation.^40–42^ The rhythm outcome in patients with persistent AF is more variable, but not much worse in some series (*Table 2*). The maintenance of sinus rhythm is not dramatically different between ‘persistent’ AF patients undergoing PVI alone compared with patients undergoing more extensive ablation approaches, including a risk to develop left atrial tachycardia with either approach (*Table 2*).^40^

**Table 2:** Controlled trials and selected observational data sets reporting sinus rhythm rates after catheter ablation of persistent atrial fibrillation

	Patients	Intervention	Control	Sinus rhythm outcome	
				Ablation	Control
Controlled trials					
Wazni^47^	70 (paroxysmal and persistent)	CA	AAD + CV	*87%*^a^	*46%*^a^
Oral^48^	146	PVI + Amiodarone	Amiodarone + CV	*74%*^a^	*58%*^a^
Stabile^49^	137 (paroxysmal or persistent)	CA: PVI + mitral line + CTI	AAD	56%	10%
Forleo^50^	70 (41 persistent)	CA	AAD + CV	*80%*^a^	*43%*^a^
Jones^51^	*52*	*CA*	*Rate control*	*88%*^a^	*n.a.*
Mont^10^	146	CA	AAD + CV	70%	44%
Verma^43^	589	PVI	PVI + lines, PVI + CFAE	59%	46%; 49%
Dong^52^	146	CA + lines (fix)	CA (stepwise)	67%	60%
Observational data sets					
Hunter (multi centre)^53^	586 (persistent)	CA (1.8 mean procedures, mainly PVI)	n.a.	*60%*^a^	n.a.
Scherr (single centre)^54^	150	CA (AF termination outcome)	n.a.	65%	n.a.
Schreiber (single centre)^55^	549	CA (stepwise approach)	n.a.	56%	n.a.
Haissaguerre^56^	103	CA (driver domains)	*65%*^a^	n.a.	

AAD, antiarrhythmic drugs; CA, catheter ablation; CV, cardioversion; PVI, pulmonary vein isolation.

^a^Numbers in italic indicate success rates without intensive ECG monitoring.

One of the most important recent studies in field of ablation of persistent AF was the Star AF 2 trial.^43^ This landmark study randomized 589 patients with persistent AF to PV isolation alone (*N* = 67 patients), to PVI plus linear ablation (*N* = 259 patients), or to PVI and ablation of continuous fractionated electrograms ablation (CFAE, *N* = 263 patients). The results of this study revealed no difference in outcomes of these three ablation strategies. After 18 months of follow-up, 59% of patients assigned to PVI alone were AF free, when compared with 49% of patients assigned to PVI plus CFAE ablation and 46% of patients assigned to PVI plus linear ablation. The lack of additional effects of CFAE ablation possibly came as less of a surprise as the lack of effects of linear lesions.^44,45^ The longer procedure duration of extended ablation procedures, associated with higher radiation exposure and possibly higher complication rates, should be considered in this context. Star AF 2 clearly supports the use of PVI without further ablation as the first-line therapy in patients with persistent AF, opening the possibility of catheter ablation of persistent AF using cryothermy balloons in the future.^46^ We propose that a group of patients with persistent AF respond as well to PVI as patients with paroxysmal AF.

## Targets for catheter ablation beyond pulmonary vein isolation

The most recent AF ablation consensus document considered PVI the ‘cornerstone’ of AF ablation.^7^ The document also stated that additional ablation strategies should be considered when ablating persistent AF, and expressed a need for sufficiently powered multicentre trials comparing different AF ablation strategies. At that time there was no consensus as to which of these ablation strategies was optimal.

Prior to the seminal description of triggers in the pulmonary veins initiating AF,^38^ several skilled groups developed different sets of linear left and right atrial lesions in an attempt to prevent AF.^57–61^ Several linear lesions, e.g. around the mitral isthmus or a ‘roof line’ connecting the ablation lesions encircling the pulmonary veins, have been re-used as relevant adjuncts to PVI in persistent AF (*Figure 2*).^54,56,62,63^ Additional lesions that have been proposed are a ‘box’ encircling the posterior left atrium including all four pulmonary veins, and a line isolating the left atrial appendage (*Figure 2*). Initial reports, often comprising procedures done in a single centre and relying on a few dozen of patients, were promising, while larger, confirmatory studies often yielded higher recurrence rates. One commonly proposed ablation strategy at the time was the ‘stepwise’ approach to ablation of persistent AF proposed and championed by the Bordeaux group.^64,65^ Like other developments in the field that were mainly developed and evaluated in a small number of centres, this approach has never gained wide-spread use. A recent publication describing the long-term outcomes of 150 patients who underwent the stepwise approach to ablation of persistent AF using the stepwise approach was somewhat sobering. Arrhythmia-free survival rates after a single procedure were 35.3 ± 3.9, 28.0 ± 3.7, and 16.8 ± 3.2% at 1, 2, and 5 years, respectively.^54^

**Figure 3:**
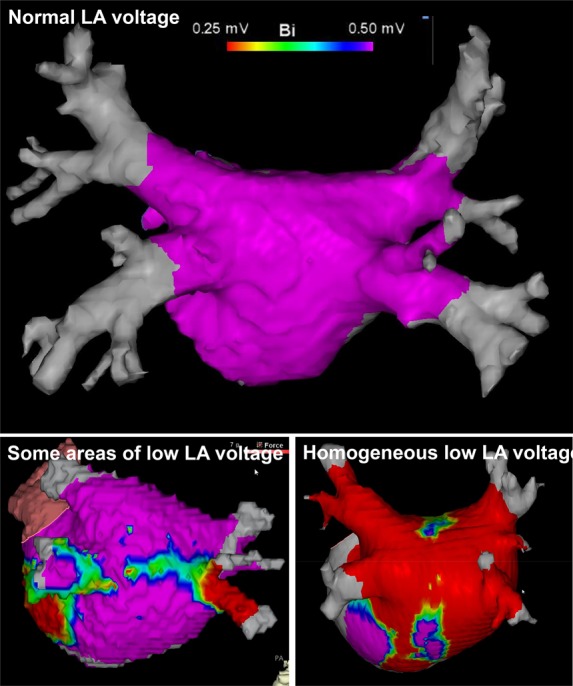
Examples of left atrial voltage maps (view onto the posterior left atrium) showing normal left atrial voltage (upper panel), confined areas of low left atrial voltage (lower left panel), and homogeneous reduction of left atrial electrogram voltage (lower right panel). Purple colour indicates areas with normal (>0.5 mV) amplitude of bipolar electrograms, red areas with low (≤0.2 mV) left atrial voltage.

New ablation strategies for ablation of persistent AF have started to emerge. One of these new strategies involves the use of a multi-electrode basket catheter to map ‘rotors’, i.e. areas that are critical for maintenance of AF.^66,67^ Some, but not all, of the published outcomes using this basket-based rotor mapping-based approach have reported encouraging results.^67,68^ Non-invasive body surface potential mapping has been used by another group to identify such critical areas (described as ‘drivers’ or ‘focal sources’ by these researchers).^69,70^ Another strategy that has been developed is to homogenize areas of scar, using MRI or voltage mapping to identify areas of scar (see *Figure 3* for illustrative examples of abnormal areas of low voltage in the left atrium). Once identified these areas of scar are ablated in an attempt to eliminate any potential re-entrant circuits.^71^ Experimental data suggest that the core of a rotor may often co-localize with areas of conduction block, in line with the behaviour of voltage vectors constructed from filtered electrograms. Hence, these two approaches may result in somewhat overlapping ablation lesions. The concept of targeting fractionated electrograms (CFAE) has been abandoned by many centres after disappointing results of controlled trials. These lesions are placed based on electrogram characteristics and do not follow a defined anatomical pattern.

Whether any of the novel strategies listed above proves to be superior to PVI alone for ablation of persistent AF remains to be determined. Currently, a large variety of ablation strategies are being employed with a goal of obtaining preliminary data concerning whether these new ablation strategies are more effective than PV isolation alone. When interpreting results from studies evaluating novel ablation strategies, it seems important to recognise a major limitation of current catheter ablation interventions: Even when the PVI is performed in selected, highly experienced centres with a clear aim to achieve complete isolation, this is only achieved in a minority of patients.^72^ Hence, better technology is needed to achieve transmural lesions. This has implications not only for the evaluation of linear lesions but also for other ablation concepts.

At some point these new ablation strategies will need to be compared head to head with PV isolation alone in sufficiently powered multicentre trials similar to the design of the Star-AF 2 Trial described above. Until that point, these new approaches must be considered experimental and their value unproven.

## Early rhythm control therapy

More and more electrophysiologists are opting to perform AF ablation early in the course of the AF journey in an attempt to reduce AF burden and limit AF-induced atrial damage. Consistent with this trend are the recommendations by both the ESC AF Guidelines and also the AHA/ACC/HRS AF consensus document that catheter ablation of paroxysmal AF may be considered as first-line therapy^11,73^ based on patient preference and when performed in experienced centres.^74^ While published trials, relying on antiarrhythmic drug therapy and often accepting interruption of oral anticoagulation after sinus rhythm restoration, have not shown a prognostic benefit of rhythm control therapy over usual care,^75^ there is biological reason to believe that sinus rhythm maintenance could help to prevent these cardiovascular events.^76–79^ Intermittent periods of sinus rhythm may reverse some of the underlying adaptive processes (‘electrical’ and ‘structural’ remodelling).^78,80^ Activation patterns in the fibrillating atria are heterogeneous and highly variable over time.^81^ The complexity of electrical activity in AF, described as drivers,^56^ epicardial break through,^82–84^ rotor,^85^ or AF cycle length,^86^ increases with longer duration of continuous AF. A novelty of the rhythm control approach in In EAST—AFNET 4 is the mandate for an early rhythm control therapy intervention,^87,88^ informed by the observation that AF-induced atrial remodelling may facilitate recurrent AF during later stages of the disease. It remains to be seen if this early intervention approach bear can also help to prevent strokes and other major cardiovascular outcomes in AF patients.

## Summary

**Figure 4:**
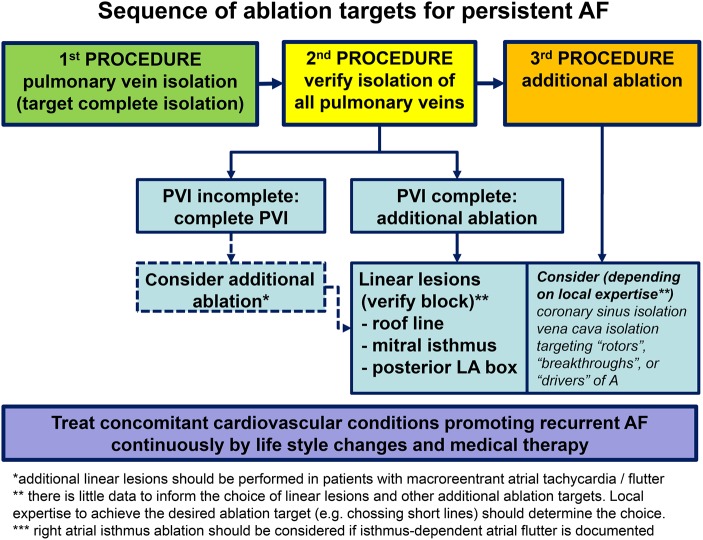
Proposed stepwise approach to catheter ablation of patients with persistent atrial fibrillation emphasizing the need to isolate the pulmonary veins before applying further ablation techniques, and illustrating the integration of medical and life style interventions underpinning the effect of catheter ablation. This proposal integrates available evidence. We recognize the need to evaluate the best AF ablation strategy in different populations of patients with persistent atrial fibrillation.

Catheter ablation is a reasonably effective intervention to achieve restoration and maintenance of sinus rhythm in patients with persistent AF. The evidence underpinning the use of catheter ablation is less strong for persistent and long-standing persistent AF than for paroxysmal AF. Acknowledging the need for further data, we suggest the following principles to guide catheter ablation of persistent AF (*Figure 4*):

1. Many patients who are clinically classified as ‘persistent AF’ will have a similar AF patterns to other patients who are classified as ‘paroxysmal AF’.
2. Catheter ablation should be considered for symptom relief in patients with persistent AF, especially after failed antiarrhythmic drug therapy.
3. Pulmonary vein isolation is a reasonable and often sufficiently effective ablation strategy in patients undergoing a first catheter ablation of persistent AF.
4. Additional ablation targets should in our view not routinely be pursued in the first procedure.
5. Optimal management of concomitant cardiovascular conditions should be an integral part of rhythm control therapy in patients with persistent AF undergoing catheter ablation.
6. Early rhythm control intervention has conceptual benefits, but needs evaluation in controlled trials. At present, it seems reasonable to not delay catheter ablation unduly after the decision for rhythm control therapy has been taken.

## What next?

There are development needs for this exciting and understudied area in clinical electrophysiology. Among them, the following appear of special relevance to us:

1. There is a need to develop better technology to achieve safe and reliable transmural lesions in the left atrium.
2. We need to identify clinical markers for different types of AF that allow to identify persistent AF patients who will benefit from catheter ablation.^89^
3. Research into reduction of ablation complications and development of simple techniques to achieve PVI^46^ is needed.
4. Several additional ablation strategies have been evaluated and abandoned, while others (including promising novel ideas) are in need of adequate evaluation in sufficiently powered controlled clinical trials, e.g. in patients with recurrent AF after successful PVI. This requires international cooperation of major ablation centres to allow the conduct of properly powered controlled trials.

## Authors' contributions

P.K. handled funding and supervision, drafted the manuscript. P.K., H.C. (literature review) acquired the data, conceived and designed the research. H.C. made critical revision of the manuscript for key intellectual content. The concept of this review article is based on a presentation given during the 2015 ESC congress in London.

## Funding

The writing of this review was partially supported by European Union [grant agreement no 633193 (CATCH ME, Characterizing Atrial fibrillation by Translating its Causes into Health Modifiers in the Elderly) to P.K.], Fondation Leducq (to P.K.), and British Heart Foundation (FS/13/43/30324 to P.K.).

**Conflict of interest:** H.C. reports personal fees from Medtronic, grants and personal fees from St Jude Medical, personal fees from Abbott Medical, outside the submitted work. P.K reports grants from British Heart Foundation and European Union, during the conduct of the study; personal fees from several industry partners, grants from several industry partners (institutional grants), grants from several public funders and charities (DZHK/BMBF, EU, BHF, and Leducq Foundation), outside the submitted work; In addition, P.K. has a patent AF therapy pending to University of Birmingham, and a patent Markers for AF pending to University of Birmingham.
